# 

**Published:** 1897-01

**Authors:** 


### CONTENTS:

### SELECTIONS.

Article I.—Filling of Canals and Pulp Cavities. By J. Foster Flagg, D. D. S. - - - - - - - 385

" II.—Some Facts About the Automatic Mallet. Dr. W. G. Laine. - - - - - - - - - - 401

" III.—Surgery of the Maxillary Sinus. By Dr. J. D. Patterson. - - - - - - - - - 404

" IV.—Simplified Treatment of Teeth With Exposed Pulps. By Withold Lindemann. - - - - - 406

" V.—Points of Interest in the Care of Children's Teeth. By B. J. De Vries, D. D. S. - - - - - 410

" VI.—When to Fill Teeth. By I. C. Edington, D. D. S. - 416

### COMMUNICATED.

Report of the Committee on National Dental Museum and Library. 419

Chicago Dental Society. - - - - - - - - 423

Odontographic Society of Chicago. - - - - - - - 424

### EDITORIAL, ETC.

Requirements to Practice Dentistry in Foreign Countries. - - 424

### MONTHLY SUMMARY.

Formalin in Dental Practice. - - - - - - - 428

Obtunding Sensitive Dentine. - - - - - - - 428

Cataphoresis Simply Explained. - - - - - - 429

Preparing a Tooth for a Porcelain Crown. - - - - - 429

Dr. Jack's Views on Implantation. - - - - - - 430

Hypnotism. - - - - - - - - - - 430

The Use of Roentgen Rays. - - - - - - - 431

Smoking and Intellectual Labor. - - - - - - 432

Foreign Dentists in Hungary. - - - - - - - 432

Temporary Stopping. - - - - - - - - 432

